# Vitamin D Is a Multilevel Repressor of Wnt/β-Catenin Signaling in Cancer Cells

**DOI:** 10.3390/cancers5041242

**Published:** 2013-10-21

**Authors:** María Jesús Larriba, José Manuel González-Sancho, Antonio Barbáchano, Núria Niell, Gemma Ferrer-Mayorga, Alberto Muñoz

**Affiliations:** Instituto de Investigaciones Biomédicas “Alberto Sols”, Consejo Superior de Investigaciones Científicas, Universidad Autónoma de Madrid, Arturo Duperier 4, Madrid 28029, Spain; E-Mails: mjlarriba@iib.uam.es (M.J.L.); jmgonzalez@iib.uam.es (J.M.G.-S.); abarbachano@iib.uam.es (A.B.); nniell@iib.uam.es (N.N.); gferrer@iib.uam.es (G.F.-M.)

**Keywords:** vitamin D, 1α,25-dihydroxyvitamin D_3_, Wnt, β-catenin, colon cancer, vitamin D receptor

## Abstract

The Wnt/β-catenin signaling pathway is abnormally activated in most colorectal cancers and in a proportion of other neoplasias. This activation initiates or contributes to carcinogenesis by regulating the expression of a large number of genes in tumor cells. The active vitamin D metabolite 1α,25-dihydroxyvitamin D_3_ (1,25(OH)_2_D_3_) inhibits Wnt/β-catenin signaling by several mechanisms at different points along the pathway. Additionally, paracrine actions of 1,25(OH)_2_D_3_ on stromal cells may also repress this pathway in neighbouring tumor cells. Here we review the molecular basis for the various mechanisms by which 1,25(OH)_2_D_3_ antagonizes Wnt/β-catenin signaling, preferentially in human colon carcinoma cells, and the consequences of this inhibition for the phenotype and proliferation rate. The effect of the vitamin D system on Wnt/β-catenin signaling and tumor growth in animal models will also be commented in detail. Finally, we revise existing data on the relation between vitamin D receptor expression and vitamin D status and the expression of Wnt/β-catenin pathway genes and targets in cancer patients.

## 1. Introduction

Vitamin D_3_ is obtained from the diet or mainly synthesized in the skin upon UV-B solar radiation. Subsequent hydroxylation in the liver, kidney and other tissues renders 1α,25-dihydroxyvitamin D_3_ (1,25(OH)_2_D_3_, calcitriol), the most active vitamin D metabolite. 1,25(OH)_2_D_3_ is a pleiotropic hormone with many regulatory effects. In addition to its classical action on intestinal calcium absorption and bone biology, 1,25(OH)_2_D_3_ inhibits proliferation, migration and anchorage-independent growth, and promotes differentiation of a variety of cultured cancer cells [1,2,3]. Consistently, numerous studies have shown tumor-suppressive actions (anti-angiogenic, anti-invasive, antimetastatic) in animal models [3], and epidemiological data suggest a protective role of vitamin D against several neoplasias, particularly colorectal cancer [4,5].

1,25(OH)_2_D_3_ regulates gene expression via binding to the vitamin D receptor (VDR), a member of the superfamily of nuclear receptors that is expressed in many normal and cancer cell types. VDR heterodimerizes with Retinoid X Receptor (RXR) and acts as a ligand-regulated transcription factor modulating the transcription rate of hundreds of target genes [1,6,7]. VDR also mediates rapid, non-genomic effects of 1,25(OH)_2_D_3_ on membrane and cytosolic signaling molecules (ion channels, kinases, phosphatases) [8]. These extra-nuclear effects are sometimes required for the gene regulatory activity of 1,25(OH)_2_D_3_/VDR complexes [9].

Human Wnts are a group of 19 secreted proteins that control proliferation, survival, migration, differentiation, and/or lineage decisions in many cell types during development and adult life [10]. To this end, Wnts activate a series of signaling pathways upon binding to specific membrane receptors: the Wnt/β-catenin or canonical pathway and several β-catenin-independent non-canonical pathways. Activation of a particular Wnt pathway depends on the individual Wnt ligand and the repertoire of receptors expressed in the target cell [11,12].

The Wnt/β-catenin pathway controls the intracellular levels of β-catenin. In the absence of Wnt signals, free β-catenin is targeted by a cytoplasmic protein complex known as the β-catenin destruction complex, which promotes its phosphorylation, ubiquitination and degradation by the proteasome. Components of this complex include the tumor suppressors AXIN and APC and the protein kinases CK1 and GSK3β. Wnt binding to membrane heterodimeric receptors (Frizzled and LRP5/6) results in inhibition of destruction complexes and subsequent accumulation of unphosphorylated β-catenin molecules in the cytoplasm, a part of which enters the nucleus and behaves as a co-activator of LEF/TCF transcription factors. β-catenin/LEF/TCF target genes encode proteins that are involved in most if not all cellular processes including proliferation, cell cycle regulation, metabolism, migration, lineage commitment, and differentiation [11,13].

The Wnt/β-catenin pathway is mainly active during embryonic development, although it also contributes to homeostasis in different tissues in adult life. Importantly, its abnormal activation in a series of epithelia is linked to generation or progression of carcinomas of the colon, breast, liver, pancreas and others. In particular, activation of the Wnt/β-catenin pathway is the initial event in a high proportion of colorectal carcinomas [11,14], and recent massive sequencing has shown that over 94% of colon tumors harbor mutations in one or more genes of the pathway [15]. Importantly, alterations in other genes and/or pathways increase the level of activation of Wnt/β-catenin signaling in colon cancer cells. Thus, crosstalk between hepatocyte growth factor (HGF)/c-MET and β-catenin signaling sustains and increases the invasive properties of colorectal cancer cells [16]. Moreover, activation of Wnt signaling in colon cancer stem cells appears to require a co-stimulatory signal involving c-MET activation by stromal-derived HGF [17,18]. Also, mutant K-RAS may enhance β-catenin/TCF-dependent transcription in APC mutant K-RAS-dependent colon cancer cells by a mechanism involving bone morphogenetic protein (BMP)-7 secretion and autocrine signaling, leading to activation of TGF-β-activated kinase (TAK1) [19].

Mutations in the tumor suppressor *APC* gene are by far the most frequent insult leading to constitutive activation of the Wnt/β-catenin pathway in colorectal cancer. However, in a small percentage of cases, mutations in *AXIN* or in the *CTNNB1*/β-catenin proto-oncogene itself have also been found. In addition, 20%–40% of hepatocellular carcinomas present mutations in *CTNNB1*/β-catenin or *AXIN* [20,21,22]. By contrast, overproduction of Wnt factors and/or repression of pathway inhibitors (SFRP, DKK-1...) have an important contribution in a proportion of hepatocellular carcinomas and other cancers such as breast or pancreas [22].

The Wnt/β-catenin pathway cannot be considered intrinsically tumorigenic, as it promotes differentiation of some cell types such as osteoblasts, myoblasts and neural precursors [23,24,25,26]. Moreover, Wnt/β-catenin signaling has controversial effects on melanoma [27] and its activation seems to collaborate with B-RAF inhibitors to impede melanoma progression [28]. Why the aberrant activation of this pathway in the colonic epithelium is so important for the initiation and progression of colon cancer is unclear. 

Non-canonical Wnt pathways include the so-called planar cell polarity pathway which involves activation of Rho small GTPases and JNK and ROCK kinases, and the calcium pathway with activation of protein kinase C, calmodulin kinase, calcineurin and NFAT transcription factor [29,30]. The role of non-canonical Wnt pathways in cancer is unclear. Several authors suggest that they antagonize the tumorigenic effects of Wnt/β-catenin signaling [31]. Others propose that while this may occur at early stages of carcinogenesis, non-canonical pathways would contribute at later stages [32].

## 2. Antagonism of Wnt/β-Catenin Signaling by 1,25(OH)_2_D_3_

The first evidences of crosstalk between nuclear hormone receptors (NHR) and Wnt/β-catenin signaling appeared the late 1990s [33] and early 2000s [34,35]. Since these initial discoveries, several laboratories have demonstrated functional interactions between both pathways underlying important biological and pathological processes. The nature of this crosstalk is complex and not fully understood, as the functional outcome depends largely upon cellular context [36,37]. In general, β-catenin potentiates NHR activity while liganded NHRs attenuate or even repress β-catenin signaling, although there are important exceptions to this rule. Molecular mechanisms underlying this crosstalk are also abundant and include, but are not limited to, physical interaction between NHRs and β-catenin or TCF/LEF transcription factors. In this review we focus on the crosstalk between VDR and Wnt/β-catenin signaling in cancer, with special emphasis on colon cancer, and the functional outcome of this interaction.

### 2.1. Studies in Cultured Cells

**Figure 1:**
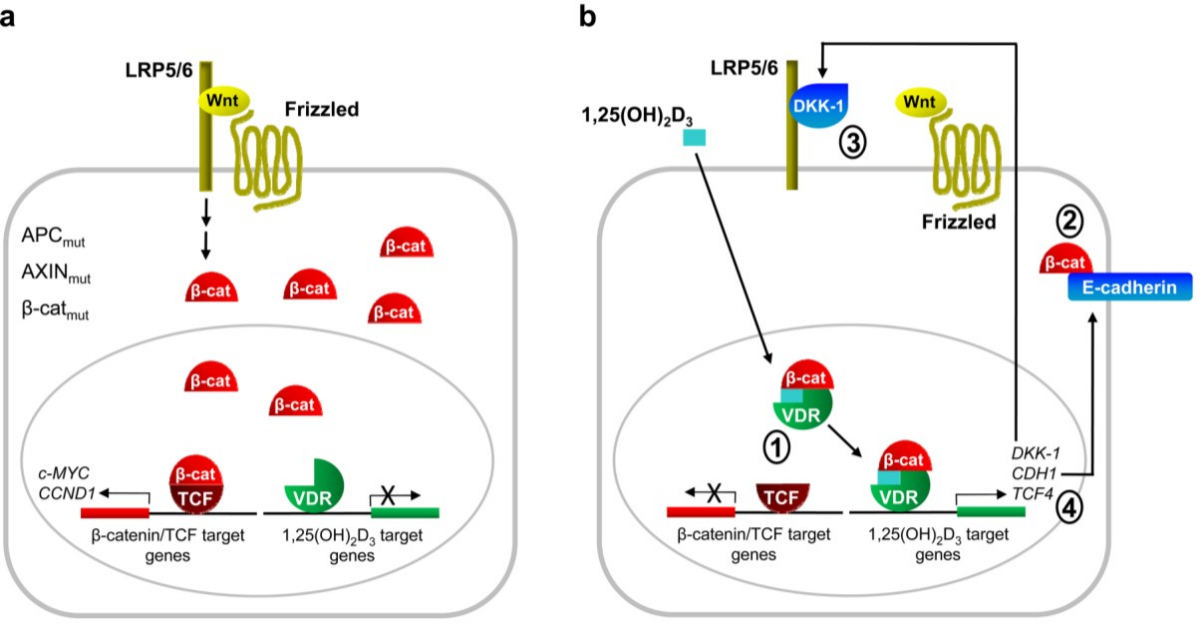
Schematic representation of the mechanisms of Wnt/β-catenin pathway repression by 1,25(OH)_2_D_3_ in human colon carcinoma cells. (**a**) Wnt/β-catenin pathway is activated by mutation of *APC*, *CTNNB1*/β-catenin or *AXIN* genes, or by deregulated signaling from Wnt plasma membrane receptors. These alterations cause accumulation of β-catenin protein in the cytoplasm and nucleus and transcription of its target genes; (**b**) 1,25(OH)_2_D_3_ inhibits β-catenin/TCF transcriptional activity in colon carcinoma cells by several mechanisms. It promotes VDR/β-catenin binding, thus reducing the amount of β-catenin bound to TCF (1); it induces the expression of *CDH1* gene coding for E-cadherin, which sequesters β-catenin at the plasma membrane *adherens* junctions (2); and it enhances the expression of the extracellular Wnt inhibitor DKK-1 (3) and of TCF4 (4). Additional paracrine mechanisms of antagonism have been proposed.

Results from our group showed that 1,25(OH)_2_D_3_ antagonizes the Wnt/β-catenin pathway in human colon cancer cells by three main mechanisms (Figure 1). First, it induces VDR/β-catenin interaction, thus reducing the amount of β-catenin bound to TCF [35]. Second, 1,25(OH)_2_D_3_ induces E-cadherin expression, leading to β-catenin nuclear export and relocation to the *adherens* junctions at the plasma membrane [35]. And third, 1,25(OH)_2_D_3_ induces the expression of Dickkopf (DKK)-1, an extracellular inhibitor of Wnt signaling [38]. As a result, we have found that 1,25(OH)_2_D_3_ inhibits the expression of several β-catenin/TCF target genes such as *c-MYC*, *TCF1*, *LEF1*, *AXIN2*, *PPARδ*, *CD44*, *ENC1* and *EPHB2* in human colon cancer cells [35,39,40]. These mechanisms of Wnt/β-catenin antagonism are completely dependent on VDR expression, as they are not observed in VDR-negative human colon cancer cells (SW480-R and SW620) or in VDR-positive cells (SW480-ADH) in which VDR expression has been repressed by Snail1 or Snail2 overexpression or by shRNA technology [35,38,39,40,41,42].

VDR/β-catenin interaction was later confirmed by Shah *et al*. in other cell types [43]. In addition, these authors characterized the domains responsible for the interaction: the *C*-terminal region of β-catenin and the activator function-2 domain of VDR. Interestingly, while 1,25(OH)_2_D_3_ antagonizes β-catenin/TCF transcriptional activity, β-catenin potentiates that of 1,25(OH)_2_D_3_/VDR [35,43,44]. Recently, Egan *et al*. have reported that wild-type APC enhances VDR/β-catenin interaction and that the VDR ligand lithocholic acid also promotes the interaction, albeit to a lesser extent than 1,25(OH)_2_D_3_ [45].

The contribution of E-cadherin induction to Wnt/β-catenin inhibition by 1,25(OH)_2_D_3_ seems to be cell-type specific, as the antagonism has also been observed in E-cadherin-negative cell lines such us LS174T [35]. Supporting the relation between E-cadherin induction and Wnt/β-catenin antagonism, we have shown that activation of the RhoA small GTPase and induction of JMJD3 histone demethylase contribute to both the induction of E-cadherin and the inhibition of β-catenin/TCF transcriptional activity by 1,25(OH)_2_D_3_ [9,46]. In addition to human colon cancer cells, Xu *et al*. observed that 1,25(OH)_2_D_3_ induces E-cadherin expression and inhibits β-catenin/TCF transcriptional activity in rat Rama-37 mammary epithelial cells [47].

DKK-1 belongs to the Dickkopf gene family that encodes secreted proteins that bind to LRP5/6 and function as extracellular inhibitors of Wnt/β-catenin signaling. DKK-1 binding to LRP5/6 blocks Wnt-Frizzled-LRP5/6 interaction and also induces the formation of a complex with another DKK receptor named Kremen that leads to LRP5/6 endocytosis [48,49]. As most colon tumors have mutations that render a constitutively active Wnt/β-catenin pathway, the importance of DKK-1 induction for Wnt/β-catenin antagonism by 1,25(OH)_2_D_3_ is unclear. However, DKK-1 seems to have antitumor effects independent of β-catenin/TCF transcriptional inhibition, as DKK-1 overexpression in *APC*-mutant colon cancer cells decreases colony formation capacity *in vitro* and tumor growth in immunodeficient mice [50]. 

DKK-4 is a weaker inhibitor of Wnt/β-catenin signaling than DKK-1. Surprisingly, we found that 1,25(OH)_2_D_3_ inhibits DKK-4 expression in human colon and breast cancer cells and that DKK-4 overexpression in human colon cancer cells increases their migratory, invasive and angiogenic capacities [51]. These results suggest that the inhibition of DKK-4 by 1,25(OH)_2_D_3_ may contribute to the antitumor effects of 1,25(OH)_2_D_3_ in colon cancer.

The use of 1,25(OH)_2_D_3_ in cancer prevention and therapy is restricted by its hypercalcemic effects at therapeutic doses. This has led to the development of several 1,25(OH)_2_D_3_ analogues that retain the antitumor actions but lack the hypercalcemic effects of 1,25(OH)_2_D_3_. We have found that 1,25(OH)_2_D_3_ analogues EB1089, KH1060, MC903, WU515, CD578 and WY1113 inhibit β-catenin/TCF transcriptional activity in a similar or even greater extent than 1,25(OH)_2_D_3_ in human colon cancer cells [35,52]. In addition, Xu *et al*. obtained similar results with the QW and BTW analogues in colon and breast cancer cells [47].

Notably, other authors have reported different mechanisms of crosstalk between 1,25(OH)_2_D_3_ and Wnt/β-catenin pathway (Figure 1). Beildeck *et al*. found that 1,25(OH)_2_D_3_ induces the expression of TCF4 in several human colon cancer cell lines by a VDR-dependent indirect mechanism [53]. However, the consequences of TCF4 induction for β-catenin/TCF transcriptional activity are not clear. The authors proposed a model whereby in normal cells, or in tumor cells that do not have nuclear β-catenin, TCF4 upregulation would enhance the repression of β-catenin/TCF target genes. In line with this, it has been reported that TCF4 inhibits growth of colon cancer cells [54]. Thus, the induction of TCF4 expression by 1,25(OH)_2_D_3_ may have a protective role in colon cancer.

Kaler *et al*. have reported a role of tumor stroma in the interplay between Wnt/β-catenin signaling and 1,25(OH)_2_D_3_ [55]. They found that tumor cells induce the release of interleukin (IL)-1β by THP-1 macrophages. In turn, macrophage-derived IL-1β inhibits GSK3β activity in colon carcinoma cells, leading to β-catenin protein stabilization and increasing β-catenin/TCF transcriptional activity. This mechanism is repressed by 1,25(OH)_2_D_3_ through the inhibition of IL-1β production in THP-1 cells and, if functional in tumor-associated macrophages, it may be another way to interfere with Wnt/β-catenin signaling *in vivo*. 

Interestingly, Meyer *et al*. have recently identified VDR/RXR and TCF4/β-catenin cistromes using chromatin immunoprecipitation followed by high-throughput DNA sequencing (ChIP-Seq) in a human colon cancer cell line [56]. They found that VDR/RXR co-occupied 1,674 sites upon 1,25(OH)_2_D_3_ treatment, most of them distal to transcription start sites. ChIP-Seq analysis also revealed 828 β-catenin and 3,161 TCF4 binding sites: after treatment with 1,25(OH)_2_D_3_ these figures decreased slightly for the former, but increased significantly for the latter. Examination of the overlap between TCF4/β-catenin and VDR/RXR cistromes indicates that the two heterodimers colocalize at 74 sites located near a limited set of genes that included *c-FOS* and *c-MYC*. These data support a direct action of both complexes at certain gene *loci* [56].

c-*MYC* is a key regulator of cell cycle progression and its expression is frequently elevated or deregulated in human cancer [57,58]. In addition, mutational and integrative analyses have emphasized the critical role of this proto-oncogene in colorectal cancer [15]. 1,25(OH)_2_D_3_ has been reported to downregulate *c-MYC* expression by two mechanisms. On the one hand, ligand-activated VDR directly represses *c-MYC* expression by binding two vitamin D response elements (VDRE) in the *c-MYC* promoter [59]. On the other, 1,25(OH)_2_D_3_ interferes with β-catenin/TCF-induced *c-MYC* transcription mediated by Wnt responsive elements (WRE) in the proto-oncogene promoter [60]. Interestingly, Salehi-Tabar *et al*. have recently demonstrated that 1,25(OH)_2_D_3_-dependent suppression of β-catenin function in a head and neck squamous cell carcinoma cell line is largely responsible for the inhibition of *c-MYC* RNA expression [61]. Moreover, they also showed that 1,25(OH)_2_D_3_ enhanced the expression of the *c-MYC* antagonist partner Mad/Mxd1 [61], further contributing to the inhibition of *c-MYC* target genes.

Although mutations in *APC*, *AXIN* or *CTNNB1*/β-catenin genes in breast cancer are rare, the Wnt/β-catenin pathway may be active in a proportion of these carcinomas according to the accumulation of nuclear β-catenin protein observed, particularly in the triple-negative and basal-like subtypes, which are highly aggressive and of poor prognosis [62,63]. Moreover, recent data indicate that WNT10B induces transcriptionally active β-catenin in human triple-negative breast cancers and predicts survival-outcome of patients with these two types of tumors [64]. Interestingly, 1,25(OH)_2_D_3_ regulates the phenotype of cultured human breast cancer cells by modulating the level and localization of cytoskeletal and adhesion proteins. Among them, 1,25(OH)_2_D_3_ represses the expression of P-cadherin, smooth muscle α-actin and α_6_- and β_4_-integrins that are myoepithelial/basal markers [65]. In line with this, *Vdr*-deficient mice express abnormally high levels of P-cadherin and smooth muscle α-actin in the mammary gland [65]. These data suggest that 1,25(OH)_2_D_3_ may protect against the triple-negative and basal-like breast cancers that are associated with poor prognosis, perhaps via the inhibition of the Wnt/β-catenin pathway.

Remarkably, the antagonism of Wnt/β-catenin signaling by the 1,25(OH)_2_D_3_ analogue paricalcitol has also been observed in other cellular systems unrelated to cancer such as vascular smooth muscle cells and renal podocytes [66,67].

### 2.2. Studies in Animal Models

Data from several types of studies using experimental animals support a protective and therapeutic effect of 1,25(OH)_2_D_3_ against several neoplasias [3,68,69]. 

Human cancer cells injected subcutaneously into immunosuppressed mice (xenografts) are commonly used as an *in vivo* approach in cancer research. Numerous studies have shown that 1,25(OH)_2_D_3_ and many analogues significantly reduce the growth of xenografts generated by tumor cells derived from several types of cancers [68,69]. We found that the 1,25(OH)_2_D_3_ analogue EB1089 inhibits the growth of xenografts generated by SW480-ADH human colon cancer cells. Remarkably, this effect is associated with induction of E-cadherin and DKK-1 expression, β-catenin nuclear export and inhibition of the expression of the β-catenin/TCF target gene *ENC1* in the xenografts [38,39,41].

Similar results were obtained when chemical carcinogens (azoxymethane or azoxymethane plus dextran sodium sulphate) were used to induce colon tumors in mice or rats. Several studies showed that tumor incidence in these models decreases following treatment with 1,25(OH)_2_D_3_ or several analogues [68]. In addition, Bissonnette’s group found that this antitumor action of 1,25(OH)_2_D_3_ is accompanied by the induction of E-cadherin expression and the repression of the β-catenin/TCF target genes *c-Myc* and *Ccnd1*/Cyclin D1 in the colonic crypts and tumors of these animals [70,71].

*Apc*^min/+^ mice are commonly used animal model for intestinal cancer. They harbour a germ line inactivating mutation in one *Apc* allele and spontaneously develop multiple intestinal neoplasias at approximately three months of age [72]. This phenotype is due to the spontaneous mutation of the remaining *Apc* allele (loss of heterozygosity) and the consequent activation of the Wnt/β-catenin pathway. Huerta *et al*. found that both 1,25(OH)_2_D_3_ and the 1,25(OH)_2_-16-ene-19-nor-24-oxo-D_3_ analogue reduce tumor load (the sum of all polyp areas) along the entire gastrointestinal tract of *Apc*^min/+^ mice [73]. Xu *et al*. confirmed these results and showed that treatment with 1,25(OH)_2_D_3_ or two of its analogues increases E-cadherin expression, reduces nuclear β-catenin levels and inhibits the expression of the β-catenin/TCF target genes *c-Myc* and *Tcf1* in the small intestine and colon of these mice [74]. However, Irving *et al*. did not find any effect of two 1,25(OH)_2_D_3_ analogues on the growth rate of colonic tumors developed in *Apc*^min/+^ mice treated with the tumor inducer dextran sodium sulphate. As mentioned by the authors, the length of treatment with the 1,25(OH)_2_D_3_ analogues or the putative loss of *Vdr* expression in the tumors might be the cause of the discrepancy with previous studies [75].

Several studies indicate that a Western-style diet that is high in fat and low in calcium and vitamin D increases the incidence of spontaneous intestinal tumors in normal mice and dramatically accelerates tumor formation in *Apc*^min/+^ mice and in other animal models for intestinal cancer [76]. In addition, this effect is reversed if the Western-style diet is supplemented in calcium and vitamin D [76]. Yang *et al*. used transcriptomic analyses to characterize the changes induced by the Western-style diet in colon epithelial cells of normal mice. They found that Wnt signaling is one of the functional categories that are significantly enriched among the genes whose expression is altered by Western-style diet and reversed to normal by calcium and vitamin D supplementation. These genes include those coding for β-catenin and for the Wnt receptors Frizzled-2 and -10 [76,77]. Similarly, Wang *et al*. observed that calcium and vitamin D supplementation abrogates the increase of β-catenin/TCF transcriptional activity and of Frizzled-5 and Ephb2 expression promoted by Western-style diet in intestinal *villi* and colon crypt cells of normal mice [78].

Genetically-modified mice have also been used to analyze the effects of VDR on carcinogenesis. *Vdr*-deficient mice do not show an increase in spontaneous cancer incidence but are more predisposed to oncogene- or carcinogen-induced breast and skin cancer and leukemia [79]. In the distal colon, these animals display hyperproliferation and an increased rate of DNA damage by oxidative stress [80,81]. Thus, two teams attempted to decipher the effects of *Vdr* gene deletion on intestinal tumorigenesis by generating *Apc*^min/+^*Vdr*^−/−^ mice [40,82]. No differences were found in the number of small intestinal and colonic tumors. However, significantly increased tumor load and number of colonic aberrant crypt foci (premalignant lesions) were observed in *Apc*^min/+^*Vdr*^−/−^ mice as compared to *Apc*^min/+^*Vdr*^+/+^. Remarkably, the lesions of *Apc*^min/+^*Vdr*^−/−^ mice showed higher β-catenin nuclear levels and expression of its targets genes *Ccnd1*/Cyclin D1 and *Lef1* than those of *Apc*^min/+^*Vdr*^+/+^ mice, suggesting that *Vdr* deletion promotes intestinal tumor growth through the activation of the Wnt/β-catenin pathway [40,82].

### 2.3. Studies in Patients

Garland and Garland were the first to suggest that vitamin D deficiency may explain the higher colon cancer mortality rates found in latitudes with low solar radiation in USA [83]. Since then, several epidemiological studies have shown that incidence and/or risk of mortality of several types of cancer (particularly colon and breast) increases in people less exposed to sunlight or UV-B radiation, with low vitamin D dietary intake, or with low 25-hydroxyvitamin D serum levels [3,84]. Few cancer intervention clinical trials using vitamin D compounds for prevention or treatment have been completed in humans. Furthermore, the results obtained have been inconclusive, inconsistent or contradictory [3,85]. Thus, to establish a cause-effect relationship between vitamin D and cancer, it is necessary to perform new large-scale and well-designed clinical studies with cancer as the primary pre-specified outcome [3,85].

At the molecular level, Ahearn *et al*. conducted a randomized, double-blinded, placebo-controlled, 2 × 2 factorial clinical trial to analyze the effect that daily supplementation of sporadic colorectal adenoma patients with vitamin D (800 IU) and/or elemental calcium (2 g) for 6 months has on the expression of APC, E-cadherin and β-catenin in crypts of the normal appearing rectal mucosa [86]. Vitamin D or calcium supplementation increased the expression of APC and reduced that of β-catenin in the differentiation zone of the crypt (the upper 40% of the crypt), which led to an increased APC/β-catenin ratio. The supplementation only with vitamin D also induced E-cadherin expression in the same region. These results support those found in cultured cells and animal models and indicate that vitamin D can modify the expression of genes related to Wnt/β-catenin signaling in humans in directions hypothesized to inhibit colon cancer [86].

VDR is expressed in the normal colon and upregulated at early stages of colon tumorigenesis (polyps, adenomas), whereas it decreases at advanced stages (carcinomas) [87,88,89,90]. Accordingly, VDR expression is associated with high tumor differentiation, absence of node involvement, and good prognosis in colon cancer [91,92]. These data suggest that advanced colon tumors with low VDR expression will probably be unresponsive to therapy with 1,25(OH)_2_D_3_ or its analogues. Our group found that the transcription factors Snail1 and Snail2 repress VDR expression and block the antitumoral actions of 1,25(OH)_2_D_3_ in cultured colon cancer cells and in xenografted mice, including the inhibition of Wnt/β-catenin signaling. In addition, *VDR* RNA expression in colon tumors inversely correlates with that of *SNAIL1* and *SNAIL2*, suggesting that these transcription factors are responsible for the downregulation of VDR found in colon cancer [39,41,42,84,93,94]. Interestingly, Snail1 protein levels and activity are induced by canonical Wnt signaling [95,96,97,98], which may constitute a mechanism to bypass the inhibitory effect of 1,25(OH)_2_D_3_ on this pathway. In addition to colon cancer, VDR downregulation by Snail factors has been also observed in osteoblasts, osteosarcoma, breast cancer and renal cells [99,100,101,102]. 

E-cadherin expression is lost during the progression of several types of carcinomas and its downregulation is frequently associated with the acquisition of invasive and metastatic properties by tumor cells. Thus, it is considered an invasion-suppressor gene [103,104]. According to data from cultured cells and animal models, we found a significant direct correlation between *VDR* and *CDH1*/E-cadherin RNA levels in human colon tumors, suggesting that VDR/1,25(OH)_2_D_3_ may contribute to the restoration of normal E-cadherin levels in human colon cancer [93,105].

Our group reported that *DKK-1* expression is frequently downregulated in human colon cancer, which suggests that *DKK-1* may act as a tumor suppressor gene in this neoplasia [106]. *DKK-1* downregulation is due to promoter methylation in 24% of advanced colon tumors (Dukes’ stages C and D) [50]. Thus, the induction of *DKK-1* expression by 1,25(OH)_2_D_3_ found in cultured cells and animal models may contribute to restore *DKK-1* expression and antitumor effects in human colon cancer. Accordingly, we found a significant direct correlation between *VDR* and *DKK-1* RNA expression in tumor biopsies from colon cancer patients [38], and Rawson *et al*. have recently reported that dietary vitamin D intake was negatively associated with *DKK-1* promoter methylation in a large cohort of human colorectal cancer patients [107]. These results suggest that the extracellular inhibition of Wnt/β-catenin signaling may contribute to the effect of 1,25(OH)_2_D_3_ against colon carcinogenesis.

We and others found that *DKK-4* is overexpressed in human colon tumors and in colon samples from patients with inflammatory bowel disease [51,108,109]. Interestingly, our group also observed a significant inverse correlation between *VDR* and *DKK-4* RNA levels in human colorectal tumors, suggesting that the regulation of *DKK-4* observed in cell lines also occurs in patients [51].

## 3. Cooperation Between VDR and Wnt/β-Catenin Signaling

Although 1,25(OH)_2_D_3_ inhibits β-catenin/TCF transcriptional activity in colon and other cancer cells, the upregulation of the Wnt/β-catenin pathway by either ligand-activated or unliganded VDR has been described in osteoblasts and keratinocytes, where it promotes bone formation and hair follicle differentiation, respectively. Thus, the interplay between Wnt/β-catenin pathway and 1,25(OH)_2_D_3_/VDR seems to depend on the cell or tissue type.

Wnt signaling promotes the differentiation of bone marrow-derived mesenchymal stem cells to bone while it represses their differentiation to other cell types, such as adipocytes [110,111]. Some 1,25(OH)_2_D_3_ effects in bone are similar to those of Wnt, suggesting a crosstalk between both pathways. Indeed, 1,25(OH)_2_D_3_ induces the expression of the Wnt co-receptor Lrp5 in mouse osteoblasts [112,113], while represses that of the Wnt inhibitors Dkk-1 and Sfrp2 in mouse bone marrow-derived mesenchymal stem cells [114]. These effects support a role of 1,25(OH)_2_D_3_ stimulating Wnt signaling in normal bone. However, the interplay between these two pathways seems to change in tumor tissue, as it has been reported that 1,25(OH)_2_D_3_ inhibits Wnt/β-catenin signaling in SaOS_2_ osteosarcoma cells [102].

Ligand-independent actions of VDR on Wnt canonical signaling have also been reported. In the skin, absence of *Vdr*, but not of 1,25(OH)_2_D_3_, results in alopecia in mice [115], and two independent groups have demonstrated that this is due, at least in part, to impaired Wnt/β-catenin signaling in keratinocytes [116,117]. In this regard, Pálmer *et al*. have shown that VDR is a Wnt effector and that β-catenin behaves as a VDR co-activator in the skin to induce transcription of genes associated with differentiation of hair follicle lineages [117]. Although this effect is largely 1,25(OH)_2_D_3_-independent, it is enhanced by the hormone. More recently, Luderer *et al*. have reported a direct interaction between VDR and LEF1 that is independent of both ligand and β-catenin, and that is required for normal canonical Wnt signaling in keratinocytes [118]. Interestingly, *Lef1* knock-out mice develop alopecia at an early age [119] and transgenic mice expressing a dominant negative Lef1 in keratinocytes also show a phenotype that resembles that of *Vdr*^−/−^ mice [120]. Therefore, although the *in vivo* significance of the VDR-LEF1 interaction is not yet clear, it may contribute to at least some of the ligand-independent effects of VDR in the skin. Similarly to what happens in bone, this interplay seems to change in the tumoral context. The development of trichofolliculomas (benign hair follicle tumors) induced by prolonged activation of β-catenin in the skin is inhibited by the 1,25(OH)_2_D_3_ analogue EB1089 [117]. In addition, undifferentiated tumors resembling basal cell carcinomas instead of trichofolliculomas are developed by β-catenin activation in *Vdr*^−/−^ mice [117]. Accordingly, keratinocytes lacking VDR present decreased E-cadherin expression, increased β-catenin/TCF transcriptional activity, and a higher proliferation rate; whereas VDR overexpression or 1,25(OH)_2_D_3_ treatment has the opposite effect [121,122]. These results suggest that 1,25(OH)_2_D_3_/VDR suppresses epidermal tumor formation by limiting the hyperproliferative actions of β-catenin in the skin. Surprisingly, epidermal β-catenin ablation cannot reduce the increased number of UVB-induced tumors developed by epidermis-specific *Vdr* knock-out mice, suggesting that VDR has β-catenin-independent anticancer functions in this model [122].

## 4. Conclusions

Evidence from a wide series of biological systems shows that inhibition of Wnt/β-catenin pathway is one of the mechanisms of vitamin D action. Wnt/β-catenin signaling has many important regulatory effects in the organism and thus, it is conceivable that the role of vitamin D is the control of an adequate level of activation of the route in each tissue and developmental stage. This most probably includes the antagonism of Wnt/β-catenin signaling in colorectal and possibly other carcinomas in which this pathway has a crucial oncogenic action. The finding that the repressive action of vitamin D takes places at different levels of the Wnt/β-catenin pathway, by distinct mechanisms, and in several cell types reinforces the importance of this regulatory action.
